# C5b-9 Deposition in Renal Transplant Biopsies Associates With Prolonged Delayed Graft Function in Kidneys Donated After Circulatory Death

**DOI:** 10.1097/TP.0000000000005815

**Published:** 2026-06-17

**Authors:** Laura W. D. Knijff, Mieke F. van Essen, Daniëlle J. van Gijlswijk-Janssen, Esther N. M. de Rooij, Jonna R. Bank, Nicole Schlagwein, Jesper Kers, Johan W. de Fijter, Rutger J. Ploeg, Cees van Kooten

**Affiliations:** 1 Division of Nephrology and Transplant Medicine, Department of Internal Medicine, Leiden University Medical Center, Leiden, The Netherlands.; 2 Center of Expertise for Lupus-, Vasculitis- and Complement-mediated Systemic diseases (LuVaCs), Department of Internal Medicine—Section Nephrology, Leiden University Medical Center, Leiden, The Netherlands.; 3 Department of Pathology, Leiden University Medical Center, Leiden, The Netherlands.; 4 Department of Pathology, Amsterdam University Medical Center, University of Amsterdam, Amsterdam, The Netherlands.; 5 Department of Pathology, Erasmus Medical Center, Rotterdam, The Netherlands.; 6 Department of Nephrology, Antwerp University Hospital, Faculty of Health Sciences and Medicine, Antwerp, Belgium.; 7 Nuffield Department of Surgical Sciences, University of Oxford, Oxford, United Kingdom.

## Abstract

**Background.:**

Ischemia–reperfusion injury is a risk factor for delayed graft function (DGF). During reperfusion, the damaged tissues and cells are exposed to blood, thereby contributing to complement activation. The aim of this study was to investigate if complement activation correlates with the duration of DGF in donation after circulatory death (DCD) kidney transplants and whether it provides additional information beyond regular histology.

**Methods.:**

In day-10 protocol, biopsies from 64 DCD recipients, complement deposition (C4d, C3d, C5b-9) was assessed by immunohistochemistry, quantified and correlated with fDGF duration (early graft function [≤7 d], intermediate [8–20 d], and prolonged duration [≥21 d]), as well as with Banff classification.

**Results.:**

C4d and C3d deposition was not different between fDGF durations. In contrast, C5b-9 deposition was higher in recipients with prolonged DGF (median 8.1%) compared with those with early graft function (median 5.6%) and intermediate DGF (median 5.6%). Kidneys from expanded criteria donors exhibited significantly higher C5b-9 deposition compared with standard criteria donors (*P* = 0.001), but no correlation with warm or cold ischemia times. Banff classification scores showed only significant associations of C4d with interstitial inflammation (i), total inflammation (ti), and arteriolar hyalinosis (ah). Similar results were found with semiquantitative complement scoring in specific kidney compartments. No significant associations were observed between C5b-9 deposition and any of the Banff scores.

**Conclusions.:**

C5b-9 deposition is increased in DCD kidneys with prolonged fDGF, representing a marker not reflected by Banff scoring. Higher C5b-9 levels in expanded criteria donors kidneys suggest that intrinsic donor factors contributes to early complement activation after transplantation.

## Graphical abstract

**Figure:**
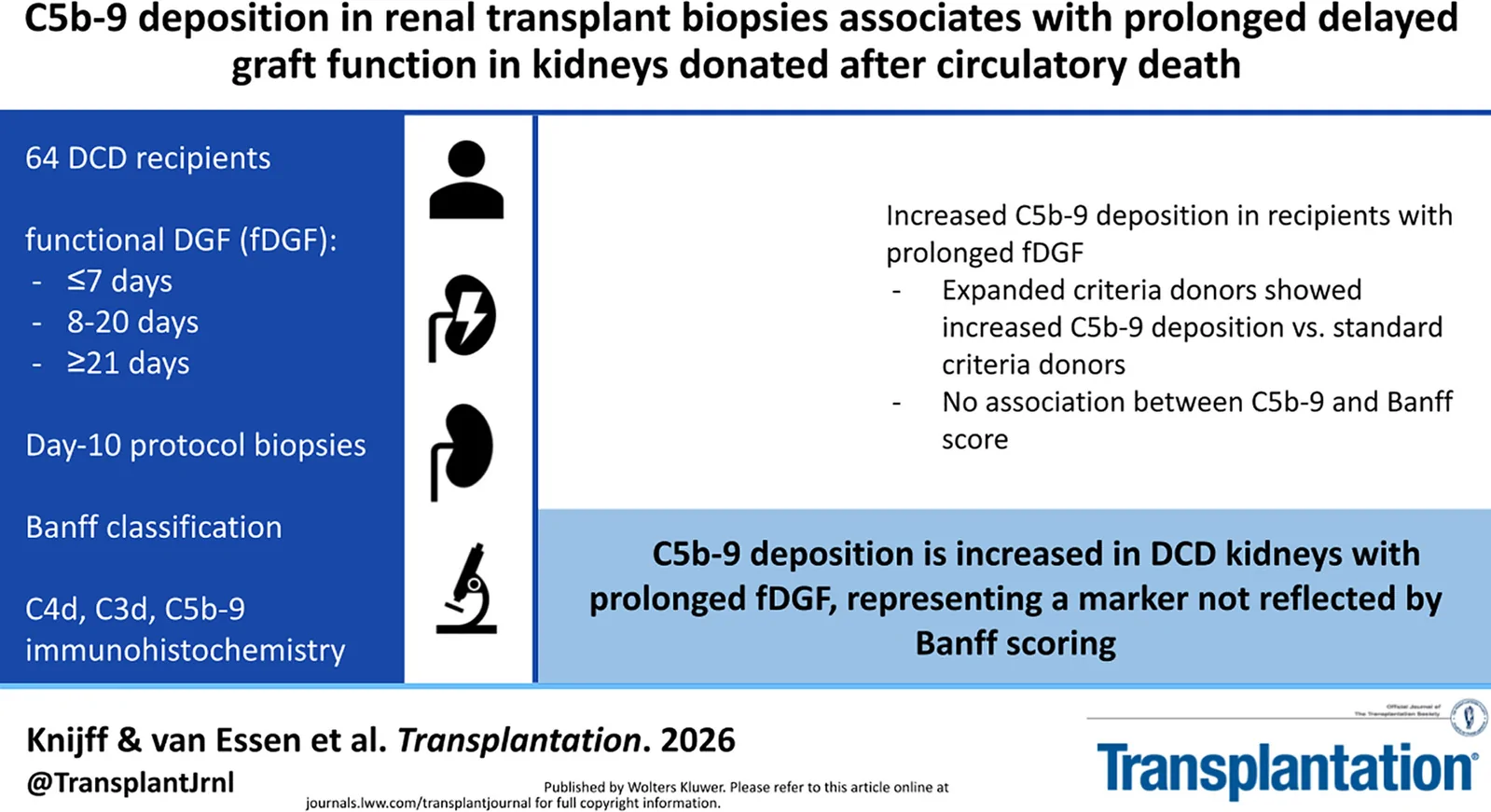

## INTRODUCTION

Renal transplantation is the gold standard for the treatment of patients with end stage renal disease. With the development of better immunosuppressive drugs, the short-term outcome of transplanted organs has improved, but long-term survival remains an issue of concern. During transplantation, the process of ischemia and reperfusion injury (IRI) is inevitable and can contribute to the onset of acute kidney injury and delayed graft function (DGF), thereby affecting graft survival.^1,2^ DGF is often defined as the need for at least 1 dialysis intervention within the first week after transplantation.^3^ However, the period of posttransplant dialysis dependency is variable and especially prolonged DGF may further impact on transplant outcomes including an inferior graft function and patient survival.^4^ The review by Swanson et al^4^ provides an important overview of the value of biopsy evaluation in prolonged DGF, but it does not address the role of complement activation. In this study, we have used the definition of functional DGF (fDGF), which is more specific and defined as the failure of serum creatinine to decrease by at least 10% daily on 3 consecutive days during the first week after kidney transplantation.^5^

Histological assessment of kidney transplant biopsies is standardized using the Banff classification, which assesses injury in different compartments with a focus on diagnostic markers of rejection.^6,7^ However, Banff focusses mainly on morphological changes and inflammatory infiltrates. Molecular mechanisms such as complement activation are not captured, except C4d deposition because of its link with antibody-mediated rejection. Additional information may help to better characterize kidney injury and identify also other potential-targeted treatment options.

The role of the complement system in IRI has been extensively studied in experimental animal models (reviewed by Santarsiero and Aiello^8^ & Delaura et al^9^). In humans, higher sC5b-9 levels have been measured via arteriovenous sampling directly after reperfusion of transplanted deceased donor kidneys, which was not observed when transplanting kidneys from living donation.^10^ Local complement activation has been observed in early and late biopsies in obtained after transplantation by deposition of C4d and C3d and has been linked with rejection.^11^ However, to date, the knowledge about the effect of local complement activation on short-term kidney function such as DGF remains limited. Therefore, a better understanding whether local complement deposition contributes to DGF may identify bespoke mechanistic targets.

Several clinical trials have focused on targeting the complement system to prevent IRI/DGF using Eculizumab and a C1 esterase inhibitor (C1-INH) as the most frequently studied treatments. C1-INH treatment has the potential to reduce activation of the classical and lectin pathway. In a phase I/II, double-blind, placebo-controlled study recipients of DCD kidneys were treated intraoperatively and at 24 h with C1-INH. C1-INH treatment resulted in significantly fewer dialysis sessions up to 2–4 wk after transplantation^12^ and post hoc analysis showed a lower incidence of graft failure after C1-INH treatment.^13^ However, the primary endpoint of reducing DGF was not achieved. Eculizumab, a potent inhibitor of C5 cleavage, prevents formation of C5b-9. Previous studies demonstrated that Eculizumab was safe and reduced posttransplant dialysis in renal transplant patients with atypical hemolytic uremic syndrome,^14^ antiphospholipid antibody syndrome,^15^ and C3 glomerulopathy.^16^ Despite these studies, several trials focusing on the effect of Eculizumab treatment on the reduction of DGF has shown disappointing results (reviewed by Golshayan et al^17^). This suggests that inhibition of the classical and lectin pathway with C1-INH may provide some beneficial effect on transplant outcomes, while inhibition of C5 with eculizumab does not appear to have the same effect. The findings raise important questions about which pathways are involved in complement activation during DGF as well as what is the time frame when complement activation is taking place.

In this study, we investigated the deposition of complement activation products in day-10 protocol biopsies from DCD kidney transplants, which is a population with a high incidence of DGF and thus well suited to assess a correlation with DGF duration. In addition, we studied whether complement activation may reveal additional characteristics beyond the typical Banff histology.

## MATERIALS AND METHODS

### Patient Population

Ninety-two human DCD recipients participated in a randomized controlled trial, the PROTECT study, which was conducted at the Leiden University Medical Center from 2005 to 2009.^18^ Characteristics of donor, recipient, transplant, and posttransplant information are described in **Table S1, SDC**, https://links.lww.com/TP/D418. In this study, 64 protocol biopsies of DCD recipients were included, whereas 28 biopsies not obtained because of withdrawal of consent (n = 7), primary nonfunction (n = 2), lack of tissue (n = 12), or technical failures (n = 7).^18^ All patients received induction therapy (anti-CD25 antibody, daclizumab, and Roche Pharmaceuticals) and maintenance therapy with cyclosporine A microemulsion (Neoral, Novartis), mycophenolate mofetil (Roche), and steroids as described before.^19,20^ The medical ethics committee of the Leiden University Medical Center approved the PROTECT study, and all patients provided written informed consent. Further details about the study are described by Aydin et al.^18^ Biopsies were scored according to the Banff classification of 2018.

### Complement Deposition in Human Renal Biopsies

Protocol biopsies obtained at day-10 were used to provide insight into early identification of histological changes that could affect graft function. Day-10 protocol biopsies of 64 DCD kidney transplant recipients were stained for components of the complement system by immunohistochemical staining. In brief, biopsies were sectioned (3 µm), acetone fixed, and frozen. All sections were thawed, and endogenous peroxidase (PBS-0.1% NaN_3_-0.3% H_2_O_2_) was blocked for 30 min at room temperature. Next, sections were stained for C4d, C3d, and C5b-9.

For C4d staining, slides were blocked in PBS-1%BSA-2% heat-inactivated normal goat serum and incubated with rabbit antihuman-C4d (1 µg/mL diluted in PBS-1%BSA (PB), BI-R4D, Biomedica, Vienna, Austria) overnight at room temperature. Next day, slides were washed (PBS) and incubated with goat antirabbit HRP (1.25 µg/mL diluted in PB, P0448, DAKO, Glostrup, Denmark) for 1 h at room temperature. Slides were washed and developed using Nova RED (SK-4800, Vector Labs, Peterborough, UK) following the protocol. Slides were washed (PBS) and counterstained with Mayers hematoxylin (1.09249.0500, Merck, Darmstadt, Germany). Slides were dried overnight at room temperature before being covered using entellan (1.07961, Merck).

Similar protocol for C3d staining was applied, except slides were blocked (PBS-1%BSA, 1 h at room temperature) and stained with rabbit antihuman-C3d (0.02 µg/mL, A0063, DAKO, in PBS-1%BSA) overnight at room temperature, followed by incubation with goat antirabbit-HRP (1.25 µg/mL, P0448, DAKO) before development.

For C5b-9 staining, slides were blocked (PBS-1%BSA, 1 h at room temperature) and stained overnight at room temperature with mouse monoclonal anti-C5b-9-DIG (AE11, HM2167, Hycult Biotech, Uden, The Netherlands, DIG-labeled in-house, 1/1500 dilution in PB). Slides were washed and incubated with sheep-FAB-anti-DIG-HRP (1/500, Roche, diluted in PB-1% heat-inactivated normal rabbit serum) for 1 h at room temperature. Slides were washed (PBS) and incubated with tyramine-FITC (1 µg/mL, prepared in-house, tyramine, T9034-4, Sigma and NHS-fluorescein, 41600, Pierce) in tyramine buffer (1 M Tris [706976, Roche], 50 mM imidazole [I0250, Sigma], pH 8.8 in distilled water with 0.003% H_2_O_2_) for 30 min at room temperature. Slides were washed (PBS) and incubated with rabbit anti-FITC-HRP (1.875 µg/mL, Abcam, in PB-1% heat-inactivated normal rabbit serum) for 1 h at room temperature. Slides were washed (PBS) and developed as described earlier. Slides were imaged (Pannoramic Midi scanner, 3DHISTECH, Budapest, Hungary) and images were analyzed (CaseViewer from 3DHISTECH).

### Quantification C4d, C3d, and C5b-9 Staining

The percentage positive area of C4d, C3d, and C5b-9 staining was determined using ImageJ image analysis software (National Institutes of Health, Bethesda, MD). In brief, digital images were loaded into ImageJ and the percentage positive area for C4d, C3d, and C5b-9 were determined via 5 representative snapshots of different areas of the biopsy obtained at 20× magnification in CaseViewer. The threshold of what was considered positive staining was manually determined for each biopsy and kept the same for the 5 snapshots. The total number of positive and analyzed pixels was calculated to determine the percentage positive area. A total of 61, 56, and 63 biopsies were available for analysis for C4d, C3d, and C5b-9, respectively. Other biopsies were excluded because of lack of tissue, or the quality of the material was insufficient. To have a semiquantitative score for different compartments, the kidney was divided into compartments for the semiquantitative analysis: glomeruli, inside the tubules, interstitia, peritubular capillaries (PTC), vessels, and the tubular basement membrane. Each compartment was scored based on the presence and intensity of the staining based on a scoring table per complement factor (**Figures S1–3, SDC**, https://links.lww.com/TP/D418). Scoring was performed by 2 independent researchers in a blinded manner, and the average score was used.

### Statistical Analysis

Statistical analysis was performed using Kruskal–Wallis test with Dunn’s multiple comparisons test. Spearman rank-order correlation test was used to determine correlations. For analyses involving multiple comparisons, *P* values were adjusted to account for the false discovery rate using the Benjamini–Hochberg procedure.^19^ Analyses were performed using GraphPad Prism v.10.2.3 (San Diego, CA). Significance was defined as *P* ≤ 0.05.

## RESULTS

### Complement Deposition in Day-10 Protocol Biopsies of Recipients of a DCD Kidney Transplant

**FIGURE 1.**
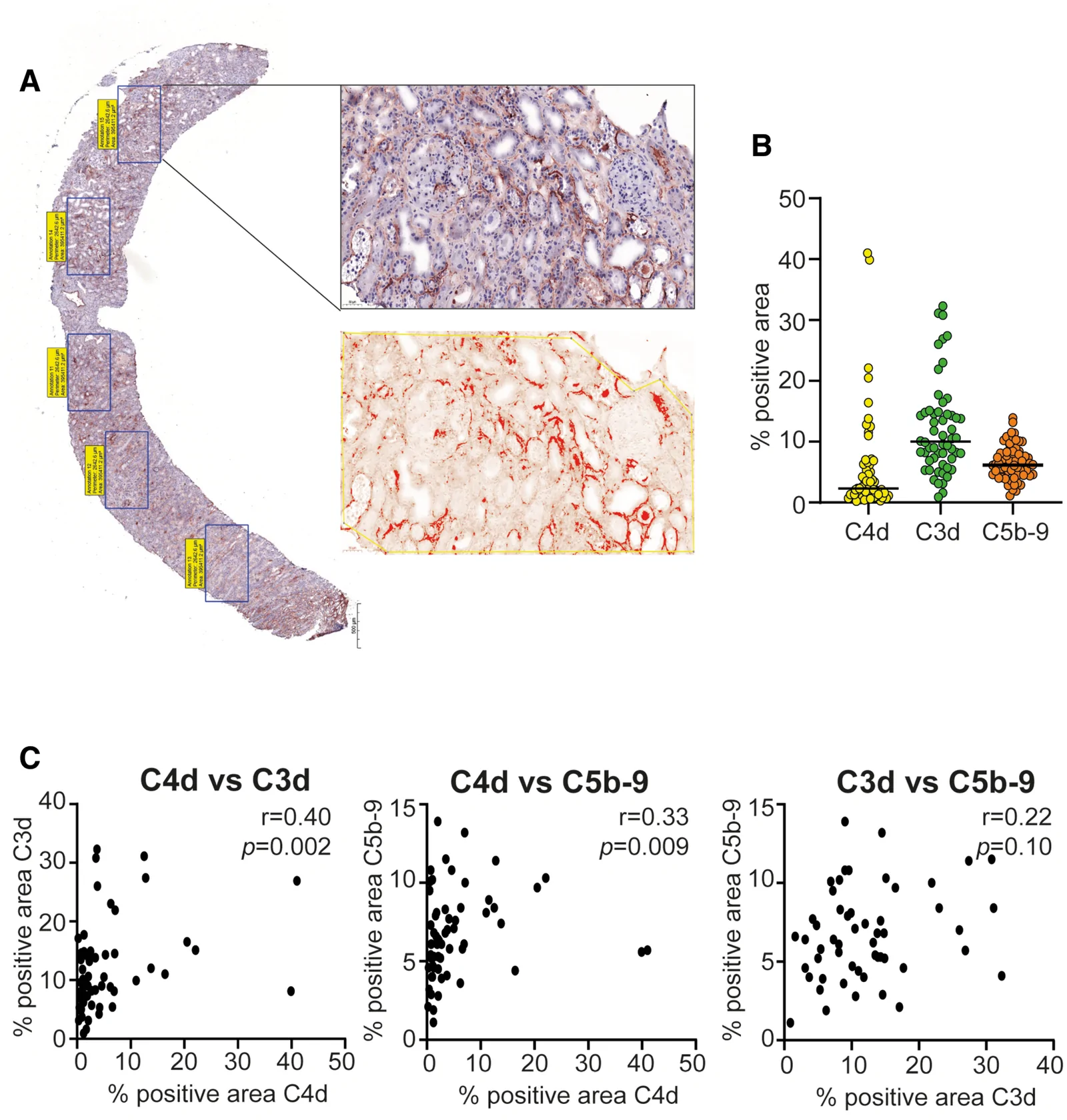
Complement deposition in day-10 protocol biopsies of DCD kidney transplant recipients. A, Representative image of complement staining analysis. Five snapshots are analyzed using ImageJ software. B, Total complement staining for C4d (n = 61), C3d (n = 56) and C5b-9 (n = 64) across all biopsies. Lines indicate the median. C, Correlations between complement factors. Spearman *r* test.

Activation of the complement system has been associated with the occurrence of DGF after renal transplantation. Here, we investigated complement activation in day-10 protocol biopsies, in a cohort of DCD grafts with a high incidence of DGF. Renal biopsies were stained for C4d, C3d, and C5b-9, representing different stages of the complement cascade, imaged, and the positively stained area was quantified using ImageJ software (Figure 1A). Both, the whole biopsy and 5 representative snapshots were used for quantification and showed significant correlations (**Figure S4, SDC**, https://links.lww.com/TP/D418). We continued analysis with snapshots to better visualize complement deposition in the different compartments. Variable levels of complement deposition were observed for C4d (median 2.3%), C3d (median 10.0%), and C5b-9 (median 6.2%) (Figure 1B). To discriminate the different complement pathways being activated, the association of different complement factors was determined. A moderate but significant association was observed for C4d and C3d (r = 0.40) and C4d and C5b-9 (r = 0.33; Figure 1C). No significant association was observed between C3d and C5b-9. This suggest that multiple pathways (classical/lectin, alternative, and terminal) are activated in the context of DGF.

### Increased C5b-9 Deposition in Recipients With Prolonged fDGF

**FIGURE 2.**
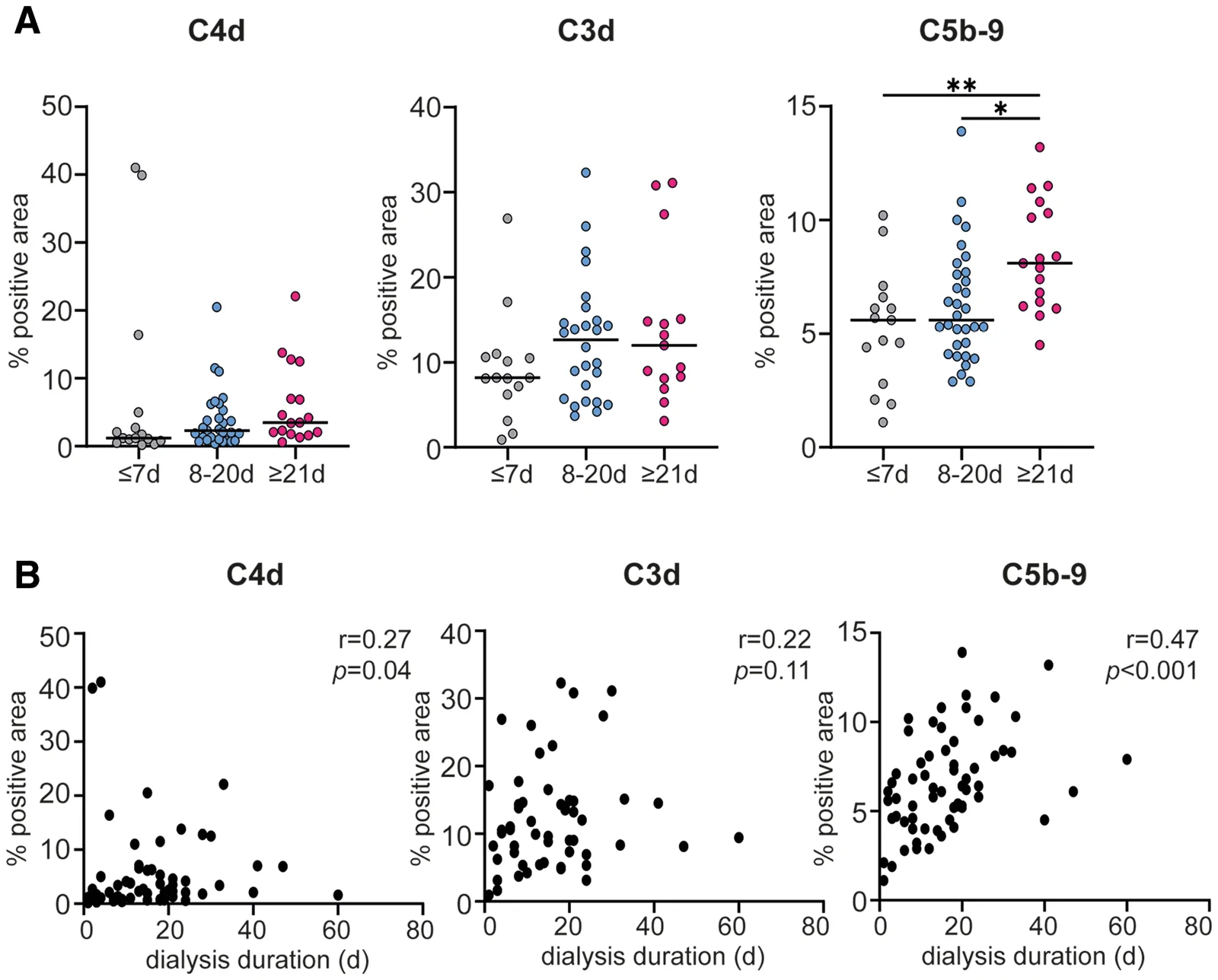
Increased C5b-9 deposition in recipients with prolonged functional delayed graft function. A) Quantification of positive staining area in 3 groups of fDGF: early graft function (≤7 d), intermediate duration (8–20 d), and prolonged duration (≥21 d). Kruskal–Wallis test. Lines indicate the median. B) Correlation between complement deposition and the dialysis duration. Spearman *r* test. *P = 0.01, **P = 0.003. fDGF, functional delayed graft function.

Next to the incidence of DGF in this cohort, the duration of DGF can vary substantially. Therefore, the positive complement staining area (area %) in these day-10 biopsies was compared with the duration of functional DGF (fDGF) and stratified into 3 groups: early graft function (≤7 d), intermediate duration (8–20 d), and prolonged duration (≥21 d). While C4d and C3d deposition showed a slight trend toward increased complement deposition in recipients with prolonged fDGF, this difference was not statistically significant (Figure 2A). C5b-9 deposition, however, was significantly higher in recipients with prolonged fDGF (median 8.1%) compared with patients with early graft function (median 5.6%) and an intermediate fDGF (median 5.6%). A similar effect was observed when complement deposition and dialysis duration were correlated as a continuous variable. C4d showed a weak but significant correlation with prolonged dialysis duration (r = 0.27; Figure 2B), while C3d showed a similar trend, but no significance was reached (r = 0.22) and C5b-9 deposition showed a moderate significant correlation with dialysis duration (r = 0.47).

### Donor Factors Affect C5b-9 Deposition

**FIGURE 3.**
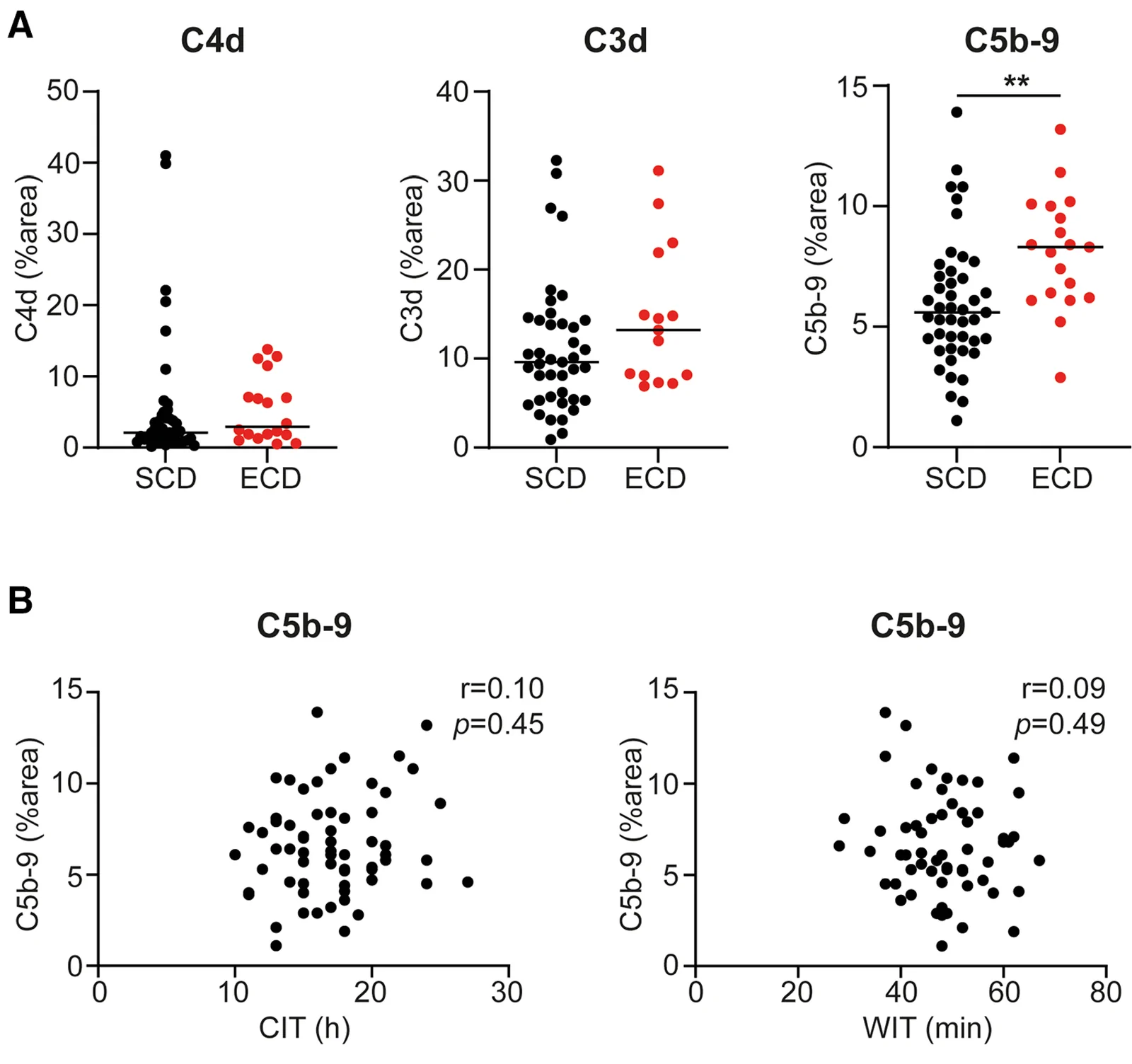
Donor factors are associated with increased C5b-9 deposition. A) Quantification of complement deposition in standard criteria donors and extended criteria donors. Lines indicate the median. Mann–Whitney test. B) Correlation of C5b-9 deposition with cold and warm ischemia time. Spearman *r* test. **P = 0.001. CIT, cold ischemia time; ECD, extended criteria donor; SCD, standard criteria donor; WIT, warm ischemia time.

To explore potential mechanisms underlying complement activation, we first assessed the effect of intrinsic donor factors. Kidneys from extended criteria donors showed significantly higher C5b-9 deposition compared with standard criteria donors (Figure 3A), while in C4d and C3d deposition such differences were not found. We then examined the relationship between complement deposition and ischemic injury, including cold ischemia time and total warm ischemia time, and found no correlations with C5b-9 (Figure 3B) or with C4d and C3d (**Figure S5A,B, SDC**, https://links.lww.com/TP/D418).

### Association of Complement Deposition With Histological Scores

Since complement deposition in these day-10 biopsies may be associated with specific histological lesions, the level of complement staining was compared with the Banff classification. As described before,^5^ >50% of biopsies scored positive for interstitial inflammation (i), tubular atrophy (ct), inflammation in sclerosed area (iifta), and vascular intima fibrosis (cv) and showed a high median score for total inflammation (ti). In addition, more than 40% showed signs of glomerulitis (g), tubulitis (t), and arteriolar hyalinosis (Table 1). C3d and C5b-9 deposition did not show significant correlation with any of the Banff classifications. In contrast, C4d deposition was found to significantly correlate with interstitial inflammation (i), total inflammation (ti), and arteriolar hyalinosis (ah) (Table 1).

**TABLE 1.** Histology

Banff classification	N (%)	C4d	C3d	C5b-9	Compartment-specific correlation
Glomerulitis (g)	26 (41)	–0.09 (0.58)	–0.22 (1.00)	0.07 (0.76)	C3d glomerulus **0.40 (0.03**)
Transplant glomerulopathy (cg)	0 (0)	n.a.	n.a.	n.a.	
Mesangial matrix increase (mm)	8 (13)	n.a.	n.a.	n.a.	
Interstitial inflammation (i)	43 (67)	**0.36 (0.02**)	0.12 (0.76)	0.10 (0.82)	C4d interstitium **0.26 (0.05**)
Tubulitis (t)	26 (41)	0.16 (0.32)	–0.08 (0.91)	0.04 (0.76)	
Total inflammation (ti)	20 (7.5–30)^*a*^	**0.38 (0.02**)	0.15 (1.00)	0.17 (0.41)	C4d interstitium **0.29 (0.03**)
Interstitial fibrosis and tubular atrophy (ifta)	13 (20)	0.21 (0.22)	–0.02 (1.00)	0.23 (0.21)	
Interstitial fibrosis (ci)	14 (22)	0.24 (0.18)	0.02 (1.00)	0.28 (0.18)	
Tubular atrophy (ct)	32 (50)	0.00 (0.99)	0.01 (1.00)	0.05 (0.82)	
Inflammation in sclerosed areas (iifta)	33 (52)	0.06 (0.73)	–0.03 (1.00)	0.10 (0.74)	
Tubulitis in sclerosed areas (tifta)	20 (31)	0.19 (0.26)	0.14 (0.77)	0.08 (0.80)	
Peritubular capillaritis (ptc)	22 (34)	0.21 (0.22)	–0.14 (0.93)	0.28 (0.24)	
Arteritis/endohelialitis (v)	1 (2)	n.a.	n.a.	n.a.	
Vascular intima fibrosis (cv)	35 (55)	0.16 (0.29)	0.00 (0.99)	0.05 (0.75)	
Arteriolar hyalinosis (ah)	26 (41)	**0.37 (0.02**)	0.18 (1.00)	0.24 (0.20)	

A total of 64 day-10 biopsies were analyzed. Shown are the number of biopsies that displayed the injury of the Banff class (score >1). Spearman r (*P* value; unless <10 cases that displayed the injury), corrected for FDR (Benjamini–Hochberg). C5b-9 deposition was not observed in the peritubular capillaries and was therefore not analyzed.

a Total inflammation (ti) is shown as median% (interquartile range).

FDR, false discovery rate.

### Compartment-specific Deposition of Complement

While the Banff classification will discriminate between specific renal compartments, the quantified area of complement deposition combined involves the total staining in the biopsy. Therefore, we performed a semiquantitative scoring of complement deposition in the different renal compartments, including the glomerulus, tubules, interstitium/tubular basement membrane, and peritubular capillaries (**Table S2, SDC**, https://links.lww.com/TP/D418) and compared these with the relevant Banff classification for that compartment. This showed that deposition of C4d in the interstitium significantly correlated with the Banff interstitial inflammation (i) score (r = 0.26) and total inflammation (ti) score (r = 0.29) (Table 1; **Table S3, SDC**, https://links.lww.com/TP/D418). Also, C3d in the glomerulus significantly correlated with the Banff glomerulitis (g) score (r = 0.40). No significant associations were observed between C5b-9 deposition and any compartment-specific Banff scores.

## DISCUSSION

Complement activation has been implicated in IRI and suggested to be a key contributor to DGF in kidney transplantation. Given the enhanced vulnerability of DCD kidney grafts to IRI because of extended warm ischemia, a better understanding of the role of complement in these grafts could help to define its role in early graft dysfunction and identify potential therapeutic targets. This study provides insight into the deposition and localization of complement activation products in recipients transplanted with a DCD donor kidney. We focused on complement activation products in day-10 biopsies, aiming to capture a more comprehensive view of the early immune responses that possibly may contribute to DGF. The deposition of complement factors C4d, C3d, and C5b-9 in the biopsies provides insight into timing and extent of complement activation as well as its potential association with graft dysfunction at a critical stage in the posttransplant recovery. C5b-9 deposition in day-10 biopsies was higher in patients experiencing prolonged fDGF (>21 d), while no difference was observed when C4d and C3d were deposited.

Our study demonstrated that complement activation was present in day-10 kidney biopsies and correlated with the duration of fDGF. This activation may originate from ischemia–reperfusion processes occurring at time of transplantation but could also already be present in the donor kidney before procurement and preservation. The observation that C5b-9 deposition correlates with donor factors rather than ischemia times suggests that intrinsic donor characteristics impact on complement activation. Future studies should identify whether complement activation is already present at earlier time points. This may be particularly relevant for extended criteria donor kidneys, that are more susceptible to complement-mediated injury. In a recent study, we have found that complement activation is already present in the DBD donor kidney.^20^ Lo Faro et al^21^ analyzed DCD donor kidney biopsies obtained during organ procurement and found that DCD kidneys with a short DGF (<7 d) showed acute cellular injury at time of donation but also result in an upregulation of repair pathways to help graft recovery. In contrast, DCD kidneys with prolonged DGF (>7 d) show reduced ability to repair and express only minimal metabolic and antioxidant responses. Our findings extend this work by showing that intrinsic donor factors, such as donor type, are associated with complement activation and the duration of fDGF, suggesting that donor factors contribute to graft injury and the extent of recovery after transplantation.

Day-10 biopsies in transplanted deceased donor kidneys with DGF are clinically important to determine whether the lack of kidney function improvement is because of acute rejection and other pathology or simply a consequence of DGF itself.^22,23^ Including C5b-9 as an additional marker could provide valuable information about the subsequent duration of fDGF, complementing information not captured by the Banff classification.

Phillips et al^24^ showed that the impact of DGF is influenced by its duration. They demonstrated that DCD kidneys with prolonged DGF (>14 d) were associated with a higher risk of death-censored graft failure and recipient mortality, while short DGF (<14 d) did not negatively impact graft or patient survival. This suggests that not DGF itself, but specifically prolonged DGF is detrimental for recovery of the kidney. Our study confirms and further expands this finding by showing that C5b-9 deposition is increased in DCD kidneys with prolonged fDGF. Therefore, we investigated whether complement deposition levels were associated with rejection and long-term kidney function. We did not observe in patients who subsequently developed rejection increased levels of complement deposition (**Figure S6A, SDC**, https://links.lww.com/TP/D418). Neither did we find a correlation with endogenous creatine clearance at year 1 (**Figure S6B, SDC**, https://links.lww.com/TP/D418) or year 5 (data not shown) after transplantation, when levels of complement deposition were divided into tertiles. Excessive complement activation—as it is present in prolonged DGF—may however affect the kidney to repair itself and recover from IRI, but whether C5b-9 is directly pathogenic or merely reflects an upstream injury cascade remains to be elucidated.

In this study, we have used a quantitative method to determine the deposition of complement factors in biopsies, with positivity indicated by the bespoke staining area %. A disadvantage of our method used for the quantification of the staining is the threshold, which must be determined for each biopsy. To prevent bias when setting these thresholds, the distinction between the 3 fDGF groups was made after finalizing the quantification. An additional limitation is the fact that we investigated kidneys transplanted after DCD donation only, and it may be interesting to extend the current studies on complement deposition in recipients after DBD donation.

By integrating these insights with previous clinical and mechanistic studies, our results support a model in which prolonged DGF reflects complement-driven injury rather than delayed recovery alone. This suggests that complement is not only a potential biomarker of a “graft at risk” but also a potential therapeutic target. Future work should therefore evaluate whether combining organ perfusion-preservation strategies with targeted complement inhibition can reduce the incidence and consequences of prolonged DGF, ultimately improving long-term outcomes in DCD kidney recipients.

Various methods have been explored to improve transplant outcomes of DCD kidneys by using hypothermic or normothermic machine perfusion.^25-27^ It is possible that machine perfusion will mitigate IRI but may have only minimal effect on complement-mediated injury. Notably, a recent study measuring complement activation proteins during normothermic machine perfusion found that high levels of C3d and C5b-9 in perfusate showed a trend toward a lower eGFR at 3 mo after transplantation.^28^ Also, high perfusate levels of C3d and C5b-9 correlated significantly with fDGF and prolonged fDGF. These findings suggest that by using machine perfusion as a platform to administer therapeutics that block complement activation could further improve transplant outcomes. Our results also show that complement activation is already present early in the deceased donation process, highlighting the opportunity to apply complement targeting strategies during machine perfusion.

In conclusion, this study shows that C5b-9 deposition is increased in DCD kidneys with prolonged fDGF. Our histological insight provides new and additional information, which is not reflected in the Banff scoring. Kidneys from extended criteria donors show increased deposition of C5b-9, suggesting an impact of the donor factors in early complement activation after transplantation. Our findings emphasize the importance of both the duration of DGF and the underlying molecular mechanisms resulting in complement activation in kidney transplantation.

## Supplementary Material


