# Supplementary material for: C5b-9 Deposition in Renal Transplant Biopsies Associates With Prolonged Delayed Graft Function in Kidneys Donated After Circulatory Death
Source: Transplantation. 2026 Jun 17;110(10):e2049–56. doi: 10.1097/TP.0000000000005815 (PMC13585161; doi:10.1097/TP.0000000000005815)
Supplement: Supplementary file 1 [file tpa-110-e2049-s001.pdf]

Supplemental digital content

Supplementary tables

Table S1. Recipient, donor and transplant characteristics

		By fDGF duration		
Characteristic	All recipients	≤ 7 days	8 - 20 days	≥21 days
n	64	15	32	17
Recipient				
Age, years	54 (± 13)	52 (± 12)	53 (± 13)	54 (± 13)
Male, n (%)	44 (69)	12 (80)	22 (69)	44 (59)
Repeated transplant, n (%)	3 (5)	1 (7)	2 (6)	0 (0)
Time on dialysis, years	4.2 (± 2.8)	4.6 (± 5.0)	4.0 (± 1.8)	4.3 (± 1.5)
Donor				
Age, years	46 (± 16)	34 (± 15)	47 (± 13)	55 (± 14)
Male, n (%)	36 (56)	7 (47)	15 (47)	14 (82)
Cause of death: CVA, n (%)	24 (38)	2 (13)	12 (38)	10 (59)
Hypertension, n (%)	14 (22)	2 (13)	7 (22)	5 (29)
Serum creatinine, µmol/L	83 (± 58)	74 (± 25)	92 (± 78)	73 (± 24)
Transplant				
HLA-mismatch class I	1.9 (± 1.1)	1.4 (± 1.0)	1.8 (± 1.0)	2.4 (± 1.2)
HLA-mismatch class II	0.9 (± 0.6)	0.7 (± 0.5)	0.9 (± 0.6)	1.1 (± 0.5)
PRA, >5%, n (%)	5 (8)	2 (13)	2 (6)	1 (6)
CIT, h	17 (± 4)	16 (± 3)	17 (± 4)	18 (± 4)
WIT I, min	17 (± 5)	17 (± 6)	17 (± 5)	17 (± 7)
WIT II, min	31 (± 8)	34 (± 6)	29 (± 7)	31 (± 9)
Post-transplant				
TFS, day-10	1.5 (± 1.2)	2.2 (± 1.3)	1.5 (± 1.1)	1.0 (± 0.9)
Serum creatinine, day-10, µmol/L	520 (± 259)	226 (± 121)	604 (± 259)	623 (± 121)
CsA trough level, day-10, µg/L	233 (± 89)	244 (± 101)	219 (± 89)	249 (± 79)
Proteinuria, day-10, g/24h	0.7 (± 0.4)	0.8 (± 0.6)	0.7 (± 0.4)	0.5 (± 0.4)

Continuous variables are presented as mean (± standard deviation). CIT, cold ischemia time; CsA, cyclosporin A; CVA, cerebrovascular accident; fDGF, functional delayed graft function; PRA, panel reactive antibodies; TFS, tubular function slope; WIT, warm ischemia time.

Table S2 Complement deposition in the kidney compartments. No C5b-9 deposition was observed in the peritubular capillaries.

	C4d	C3d	C5b-9
Glomerulus	1.0 (1.0-2.0)<br>N=43	1.0 (0.8-1.5)<br>N=41	1.5 (1.0-2.0)<br>N=44
Peritubular capillaries	1.0 (0.5-1.0)<br>N=59	2.0 (1.5-2.5)<br>N=52	0
Tubuli	1.0 (0.5-1.5)<br>N=60	1.0 (1.0-2.0)<br>N=50	1.5 (1.0-2.0)<br>N=58
Vessels	1.8 (1.0-2.0)<br>N=28	1.0 (1.0-1.0)<br>N=33	2.0 (2.0-2.5)<br>N=33
Interstitium/Tubular basement membrane	1.0 (0.0-1.0)<br>N=60	1.5 (1.0-1.5)<br>N=54	2.0 (2.0-2.5)<br>N=59

Table S3 Correlation complement deposition in different renal compartments vs. Banff classification. Spearman r, corrected for FDR. Indicated are r-value (*p*-value). N.a. not calculated because no variation was observed (all zero).

Renal compartment	Banff classification	C4d	C3d	C5b-9
Glomerulus	Banff g	-0.11 (0.48)	**0.40 (0.03)**	-0.00 (0.99)
	Banff ti	0.20 (0.19)	0.12 (0.44)	-0.28 (0.06)
Tubules	Banff t	0.05 (0.68)	0.09 (0.62)	0.00 (0.98)
	Banff ti	0.05 (0.70)	0.19 (0.19)	0.11 (0.40)
Interstitium/Tubular Basement Membrane	Banff i	**0.26 (0.05)**	0.11 (0.44)	-0.09 (0.51)
	Banff ci	0.22 (0.09)	0.25 (0.07)	0.25 (0.05)
	Banff ti	**0.29 (0.03)**	0.15 (0.29)	-0.06 (0.64)
Peritubular Capillaries	Banff ptc	0.06 (0.64)	-0.02 (0.89)	n.a.
	Banff ti	0.24 (0.07)	0.24 (0.09)	n.a.

Supplementary Figures

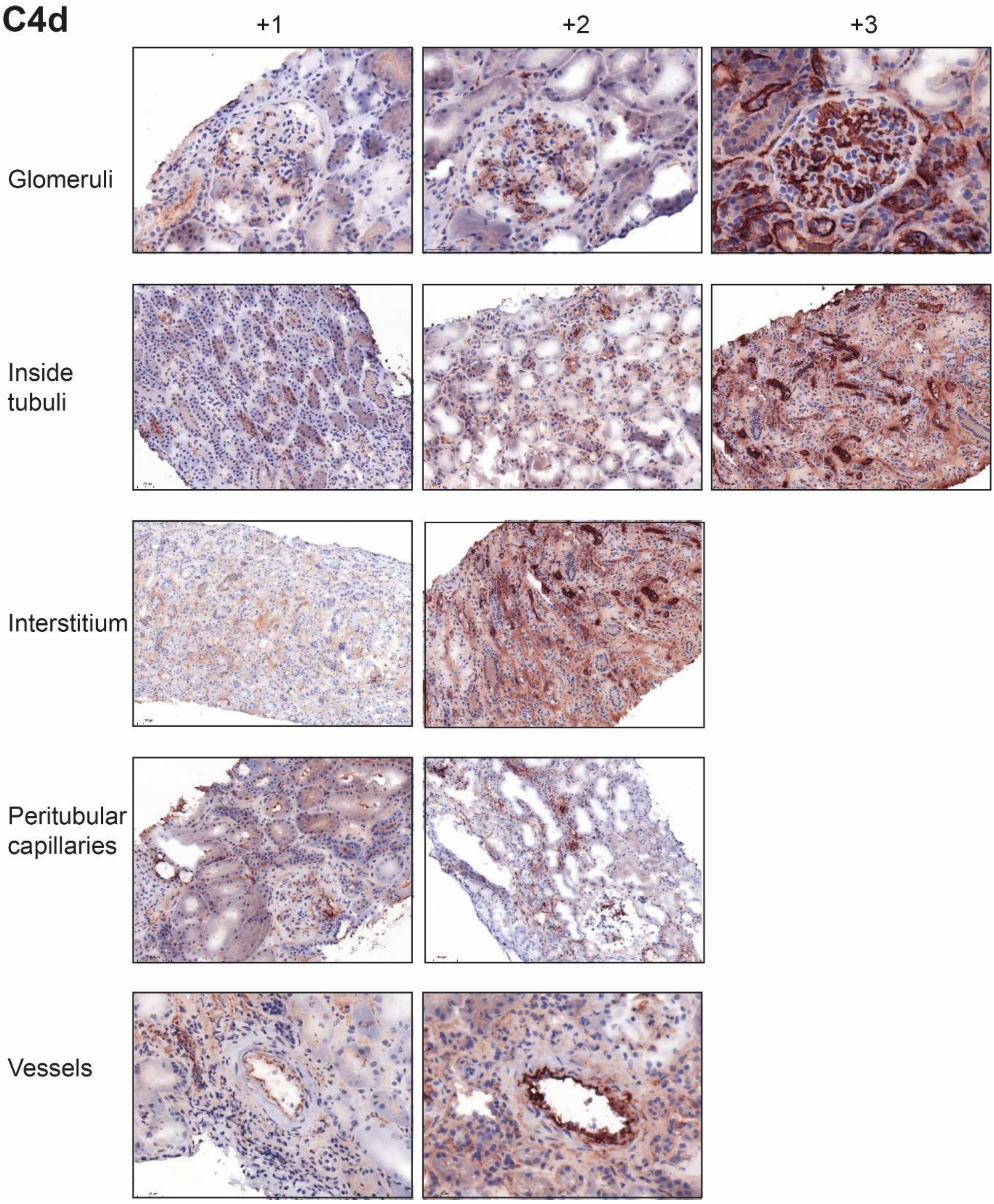

Figure S1 Scoring table for semiquantitative analysis of C4d deposition. Representative images illustrate examples of scores.

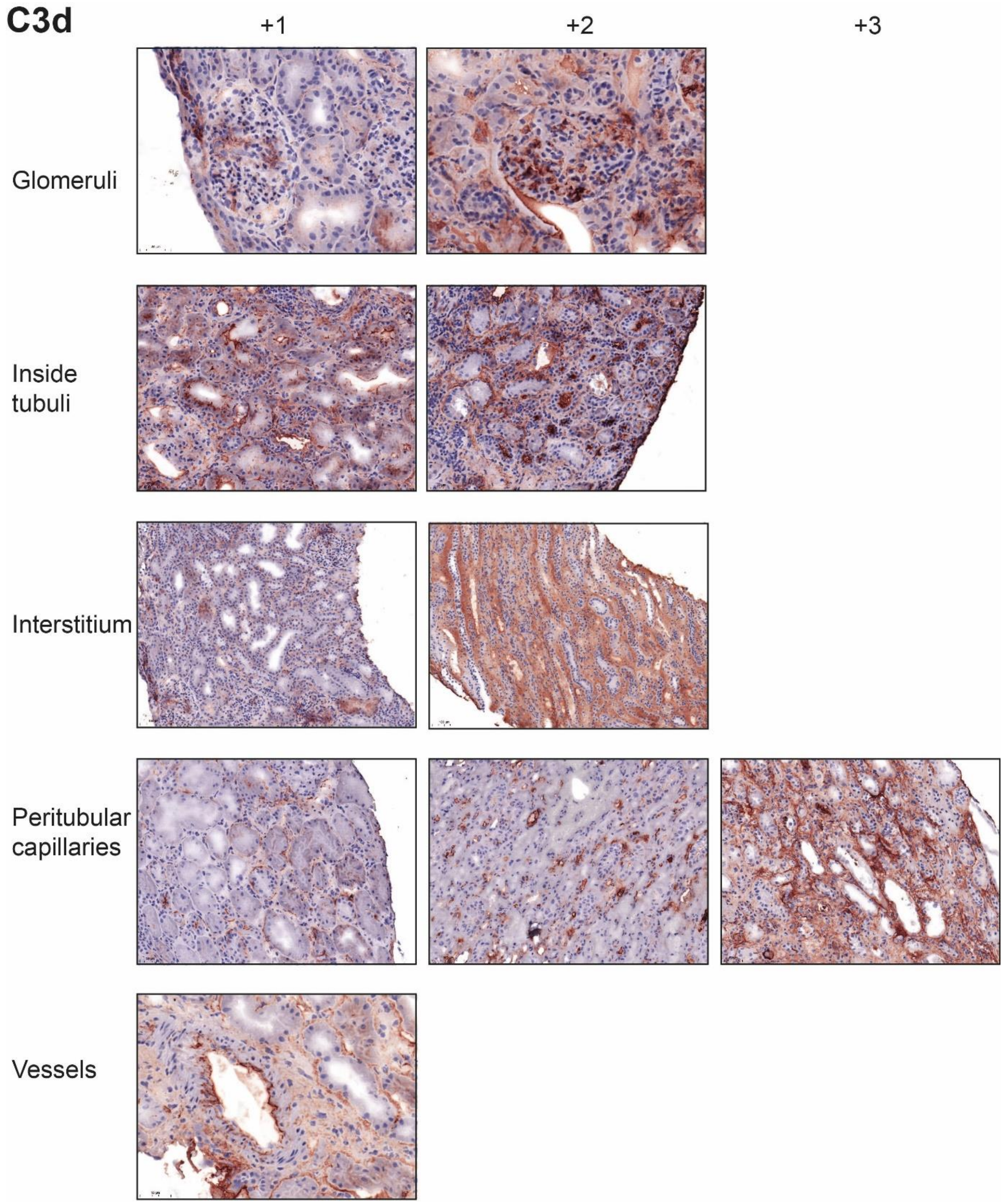

Figure S2 Scoring table for semiquantitative analysis of C3d deposition. Representative images illustrate examples of scores.

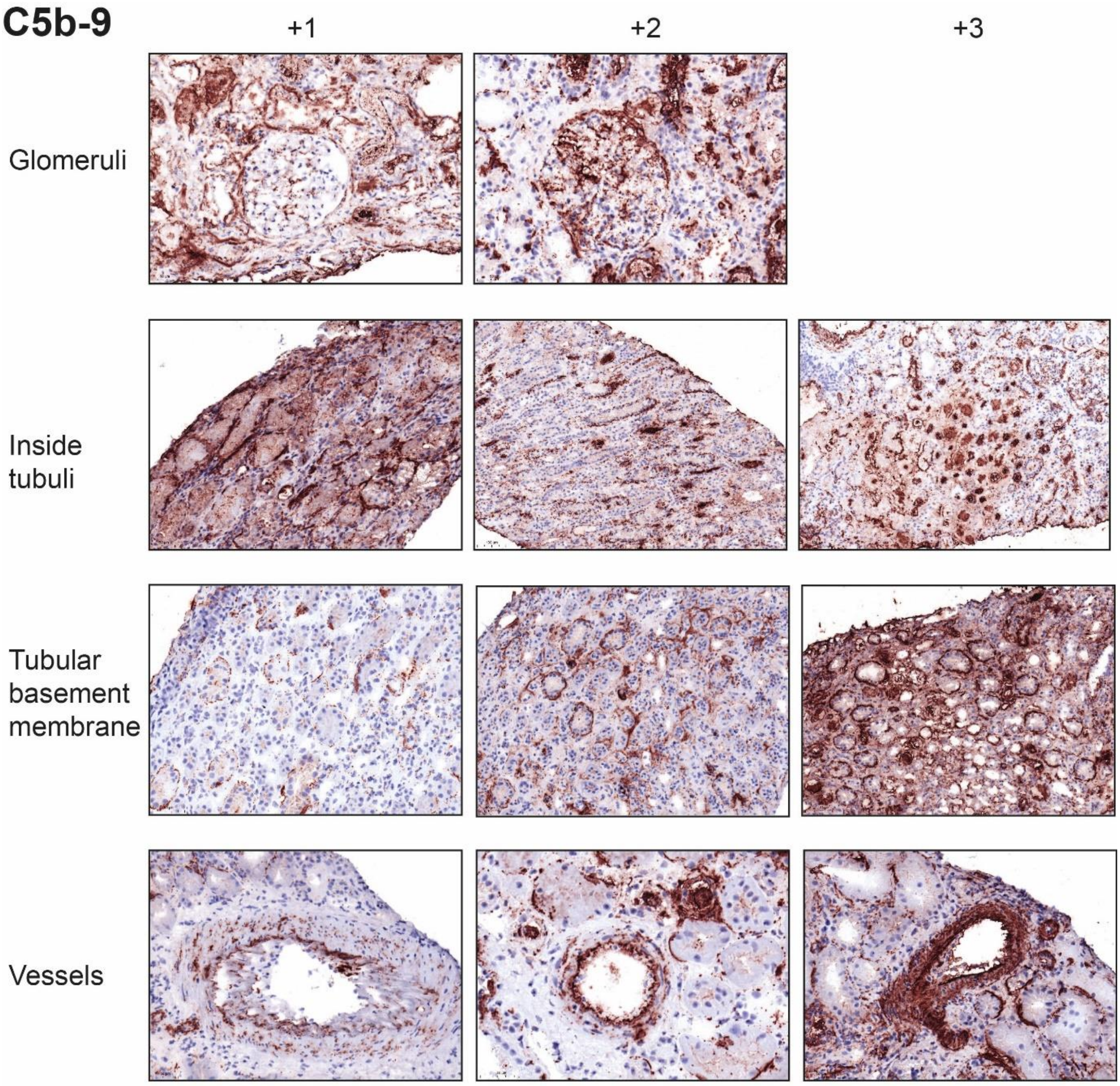

Figure S3 Scoring table for semiquantitative analysis of C5b-9 deposition. Representative images illustrate examples of scores.

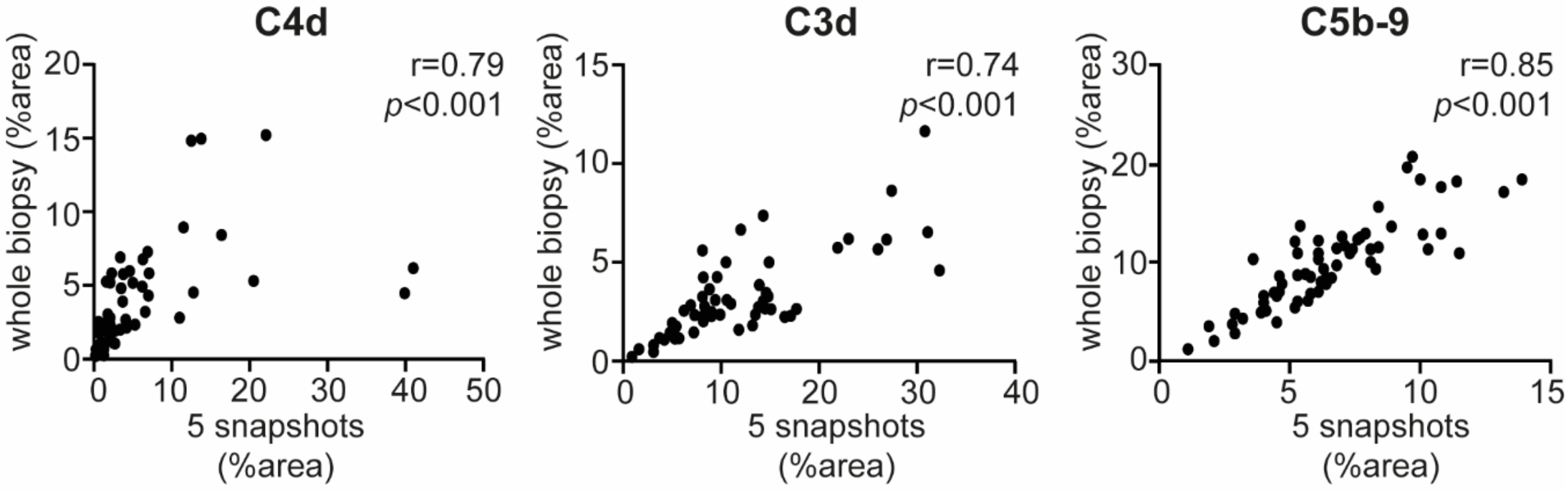

Figure S4 Correlation between whole-biopsy and snapshot-based quantification of complement deposition. Correlation between the percentage of positively stained area quantified on the entire biopsy section and on five representative high-power snapshots for C4d, C3d, and C5b-9. Spearman r test.

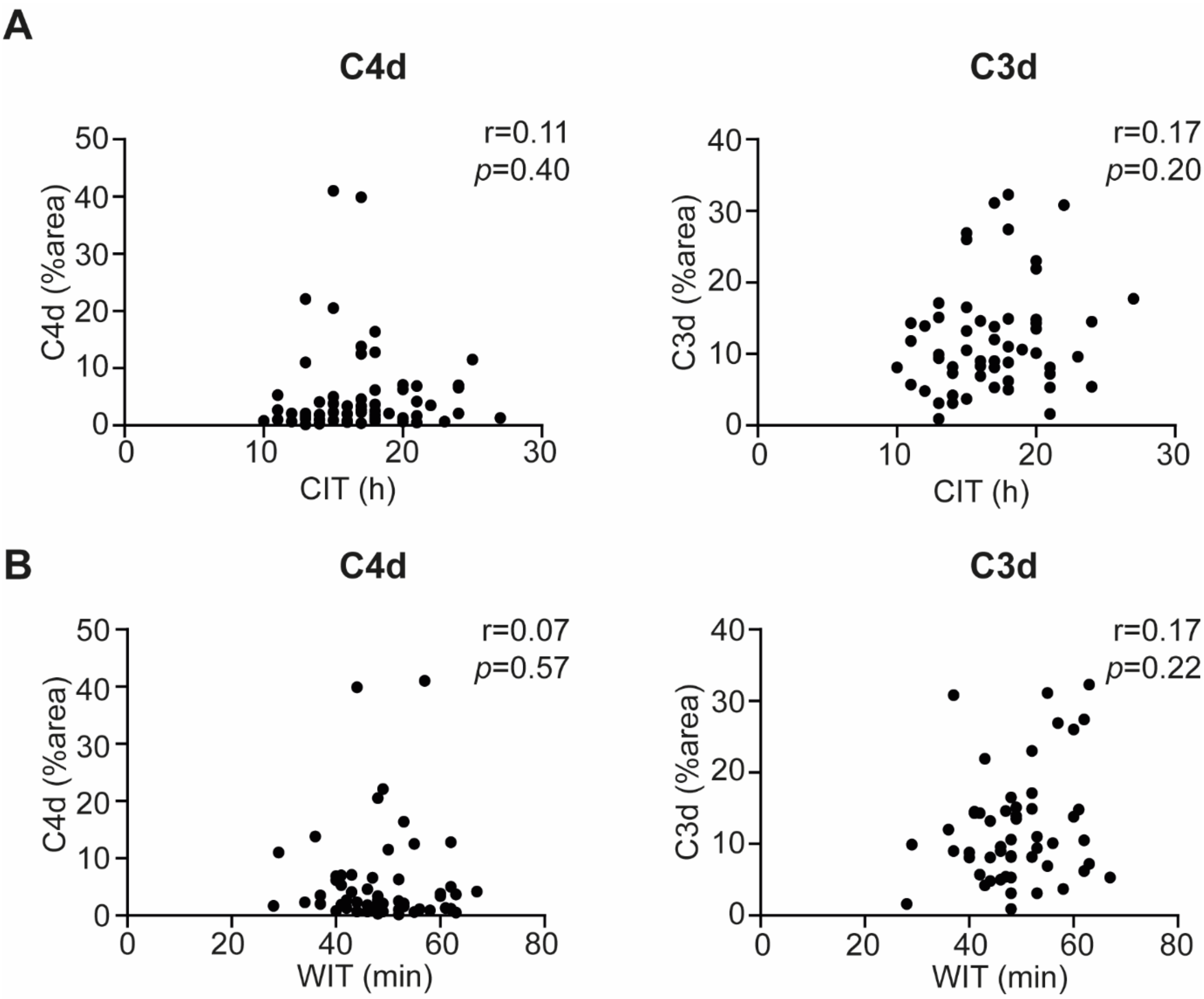

Figure S5 Cold and warm ischemia times are not associated with C4d or C3d deposition. Correlation between (A) cold ischemia and (B) warm ischemia times for C4d and C3d. Spearman r test. CIT=cold ischemia time, WIT=warm ischemia time.

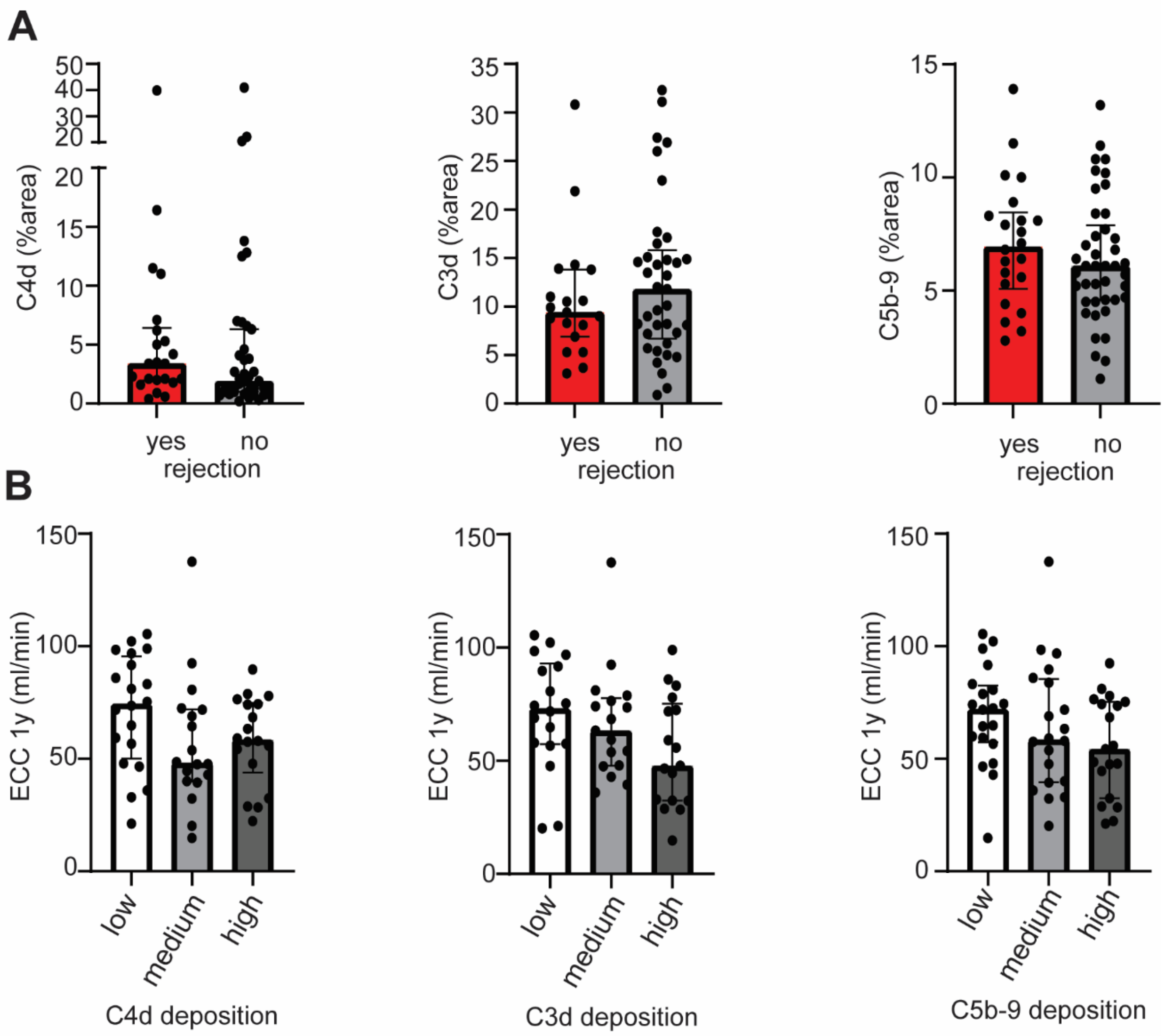

Figure S6 Association of Complement Deposition with Graft Function and Rejection. (A) Complement deposition levels (% area) in recipients with and without rejection. (B) Endogenous creatinine clearance (ECC) at 1 year after transplantation according to tertiles of complement deposition (C4d, C3d and C5b-9). Data are shown as median ± interquartile range. Kruskal-Wallis and Mann-Whitney test. ECC=endogenous creatine clearance.
